# Disease burden in Brazil and its states. Estimates from the Global Burden of Disease Study 2019.

**DOI:** 10.1590/0037-8682-0622-2021

**Published:** 2022-01-28

**Authors:** Deborah Carvalho Malta, Valéria Maria de Azeredo Passos, Ana Maria Nogales Vasconcelos, Mariângela Carneiro, Crizian Saar Gomes, Antônio Luiz Pinho Ribeiro

**Affiliations:** 1 Universidade Federal de Minas Gerais, Escola de Enfermagem, Departamento de Enfermagem Materno-Infantil e Saúde Pública, Belo Horizonte, MG, Brasil.; 2 Universidade Federal de Minas Gerais, Faculdade de Medicina, Belo Horizonte, MG, Brasil.; 3 Universidade de Brasília, Departamento de Estatística, Brasília, DF, Brasil.; 4 Universidade Federal de Minas Gerais, Departamento de Parasitologia, Belo Horizonte, MG, Brasil.; 5 Universidade Federal de Minas Gerais, Faculdade de Medicina, Departamento de Medicina Preventiva e Social, Programa de Pós-Graduação em Saúde Pública, Belo Horizonte, MG, Brasil.

To guarantee the well-being of the population of a country, it is necessary to understand the magnitude and distribution of the main health problems. The estimations of disease burden play a key role in allowing managers to perform preventive and healthcare actions guided by scientific evidence^1^. Since 2014, the Global Burden of Disease Study (GBD)-Brasil Network has been analyzing morbidity-mortality in Brazil and its states, thanks to collaboration between the Ministry of Health and the international initiative of the GBD Study powered by the Institute of Health Metrics and Evaluation (IHME) of the University of Washington^2^.

In addition to inform the estimates to policymakers, the GBD-Brasil Network collaborators have the mission of spreading this initiative into the academic realm. In 2017, the first publication of the results of this venture sought to attain visibility among researchers from the field of public health^3^. The second supplement, published in 2019, aimed to advocate the health metrics analysed by the GBD-Brasil Study^4^.

This third supplement in the *Journal of the Brazilian Society of Tropical Medicine* focuses on the study of the diseases that depict social inequality within the country. The articles’ diversity of themes reflects the triple disease burden in Brazil, illustrated through the concomitance of infectious and chronic diseases, and external cause.

The COVID-19 pandemic was a forewarning of the importance of the combined and systematic action of epidemiological surveillance and healthcare management. IHME projections, already in the end of 2020, warned of the risk of increases in mortality rates if the country did not adopt the proposed measures of physical distancing and the use of masks^5^. In this supplement, one article warns not only of the 22% of excess mortality in 2020 throughout the country, but also points out the higher mortality rates among men (25.2%) than among women (19.0%), as well as the higher rates among blacks (27.8%) than among whites (17.6%). The investigation of COVID-19 in Belo Horizonte City, also in 2020, showed that the fear of COVID-19 infections was a probable cause for the decline in hospitalizations by cardiovascular diseases, highlighting the importance of measures to mitigate the indirect effects of the pandemic.

Another two articles show that, despite the reduction in the infectious and maternal disease burden in the country, there is still much to be done to reach the levels of developed countries. Since 1990, a substantial decline in maternal mortality has been observed, but the number of deaths is still excessive, mainly due to the burden of hypertension during pregnancy, a cause that is preventable by public health actions. The positive impact of public health measures is also demonstrated in the article that shows an increase in the diagnosis and antiretroviral treatment of HIV infection, but the authors warn of the need to intensify the prevention measures among young men that have sex with other men.

Different faces of the burden caused by chronic diseases were shown. While one article describes the high premature mortality caused by the four main chronic diseases, another highlights the high burden of incapacity due to lower back pain, osteoarthritis, rheumatoid arthritis, and gout in Brazil. Other studies show the stability of incidence and mortality rates for prostate cancer since 1990, with a reduction in mortality rates in the states of greater socioeconomic development, while pancreatic cancer shows an increase in mortality rates in all Brazilian states since 2000, primarily in the elderly.

As a result of the greater visibility of GBD studies in the country, the GBD-Brasil Network was enhanced by researchers in oral health, who were responsible for three articles. One article shows that, in 2019, nearly 100 million Brazilians presented at least one oral health disorder, such as tooth loss, periodontitis, and dental caries. Another two articles discuss the burden and determining factors of the decline in incidence and mortality caused by head and neck, lip, oropharynx, and larynx cancers, which still present worrisome rates among men.

Understanding the burden of the diseases that can be attributed to the main modifiable risk factors is crucial to establish prevention and control strategies. In this supplement, one article shows the increased risk attributable to the metabolic and behavioral factors, mainly obesity and alcohol consumption, both in mortality as well as in the disability-adjusted life years of diseases in Brazil. We also present an investigation highlighting the suboptimum diet among the Brazilian population, mainly because of a diet high in red meat and sodium, and low in whole grains, while another one points out the urgency of the implementation of legal measures to reduce sodium contents in industrialized foods, since this consumption was the third higher dietetic risk for chronic diseases in Brazil. Despite the reduction in the mortality-morbidity by cardiovascular diseases and smoking since 1990, we can see in one article that risk factors for diabetes and hypertension continue high and could explain 83% of the mortality by cardiovascular diseases in 2019. We can also read that, in addition to the metabolic factors, physical inactivity is a risk factor attributable to a greater burden due to strokes in Brazil.

The burden of violence in the country is discussed in five articles with different focuses. The mortality rates by feminicide in Brazil, despite the policies of women’s rights implemented since the early 2000s, have not only remained stable since 1990, but have also continued at alarming levels, not reaching the reduction goals set forth by the UN’s Sustainable Development Agenda 2030. In addition, distant from the goal of a 50% reduction by 2030 is mortality in traffic, which remains higher in men and young adults. Despite the decline in the mortality rate in traffic, there was a significant increase in the deaths of motorcyclists. Brazil also has the eighth highest burden due to cocaine abuse in the world, with dependency rates of 2.7-fold higher among men. Men also present the highest suicide rates in the country, as demonstrated by two articles on this theme. Despite the tendency towards a drop in mortality rates by suicide throughout the country, this decline is minimal among the elderly, with a predominance of death among elderly men and major inequality throughout the states of Brazil.

Finally, we wish to highlight that the descriptions of the disease burden in Brazil illustrate, in a wide range of nuances, the importance of implementing measures to reduce socioeconomic inequality and strengthen Public Health and health research in Brazil.
